# Accuracy of the urine UCA1 for diagnosis of bladder cancer: a meta-analysis

**DOI:** 10.18632/oncotarget.16473

**Published:** 2017-03-22

**Authors:** Xiangrong Cui, Xuan Jing, Chunlan Long, Qin Yi, Jie Tian, Jing Zhu

**Affiliations:** ^1^ Pediatric Research Institute, Children's Hospital of Chongqing Medical University, Ministry of Education Key Laboratory of Child Development and Disorders, Chongqing, China; ^2^ Reproductive Medicine Center, Children's Hospital of Shanxi and Women's Health Center of Shanxi, Affiliate of Shanxi Medical University, Taiyuan, China; ^3^ Clinical Laboratory, Shanxi Province People's Hospital, Affiliate of Shanxi Medical University, Taiyuan, China; ^4^ China International Science and Technology Cooperation Base of Child Development and Critical Disorders, Chongqing, China; ^5^ Chongqing Key Laboratory of Pediatrics, Chongqing, China; ^6^ Cardiovascular Department (Internal Medicine), Children's Hospital of Chongqing Medical University, Chongqing, China

**Keywords:** UCA1, biomarker, bladder cancer, noninvasive diagnosis, urinary marker

## Abstract

Urine UCA1 has been reported as a potential novel diagnostic biomarker for bladder cancer in several studies, but their results are inconsistent. As a result of this, a diagnostic meta-analysis to assess the diagnostic performance of urine UCA1 in detecting bladder cancer was conducted. A systematic electronic and manual search was performed for relevant literatures through PubMed, Cochrane library, Chinese Wan Fang and the China National Knowledge Infrastructure (CNKI) databases up to December 30, 2016. The quality of the studies included in this meta-analysis was assessed using the Quality Assessment of Diagnostic Accuracy Studies (QUADAS-2) tool. All analyses were conducted using stata12.0 software. Six studies collectively included 578 bladder cancer patients and 562 controls met the eligible criteria. The overall diagnostic accuracy was measured by the following: sensitivity 0.81 (95% CI = 0.75-0.86), specificity 0.86 (95% CI = 0.73-0.93), positive likelihood ratio 5.85 (95% CI = 2.72-12.57), negative likelihood 0.22 (95% CI = 0.15-0.32), diagnostic odds ratio 27.01 (95% CI = 8.69-83.97), and area under the curve 0.88 (95% CI = 0.85-0.91). Meta-regression analysis suggested that ethnicity significantly accounted for the heterogeneity of sensitivity. Deeks’ funnel plot asymmetry test (*P* = 0.33) suggested no potential publication bias. According to our results, urine UCA1 has greater diagnostic value in diagnosing bladder cancer, however further research studies with more well-designed and large sample sizes are required to confirm our findings.

## INTRODUCTION

Bladder cancer (BC) is the second most common urogenital malignancy, with 50% recurrence rate and 15-40% growing into muscle invasive disease [1, 2]. Early diagnosis and reliable follow-up for recurrences after conservative treatment are extremely important for improving treatment of BC [3–5]. Cystoscopy is currently considered the golden standard for diagnosing bladder cancer, often combined with urinary cytology [6]. However, the invasive nature of cystoscopy and low sensitivity of cytology restrict their application in the early diagnosis of BC [7, 8]. Therefore, exploring more reliable non-invasive detection of new or recurrent bladder cancer is the need of the hour.

Long non-coding RNAs (lncRNAs) are a class of transcribed RNA molecules with more than 200 nucleotides in length and lack protein coding function [9–11]. Accumulating studies have shown that the dysregulation of lncRNAs was closely related to oncogenesis, metastasis, and prognosis in cancers [12–14]. Their expression patterns in various cancer types have been extensively identified, and many of these lncRNAs might be used as independent biomarkers for tumor diagnosis and treatment [15–17]. Recently, the lncRNAs, urothelial carcinoma associated 1 (UCA1), was firstly identified in the tissue and urine in 2006 and was found that it might play a crucial role in BC progression and embryogenesis [18]. In addition, UCA1 was also detected as a very sensitive and specific urine marker in BC diagnosis [19, 20].

Despite many studies have demonstrated the potential of urine UCA1 as a novel diagnostic marker for BC, the previous studies have been limited by relatively small sample size recruited in the individual studies, and no previous published meta-analysis have addressed this research question. Thus, we carried out this meta-analysis to review and assess the overall diagnostic test accuracy of UCA1 for BC diagnosis.

## RESULTS

### Study characteristics

**Figure 1:**
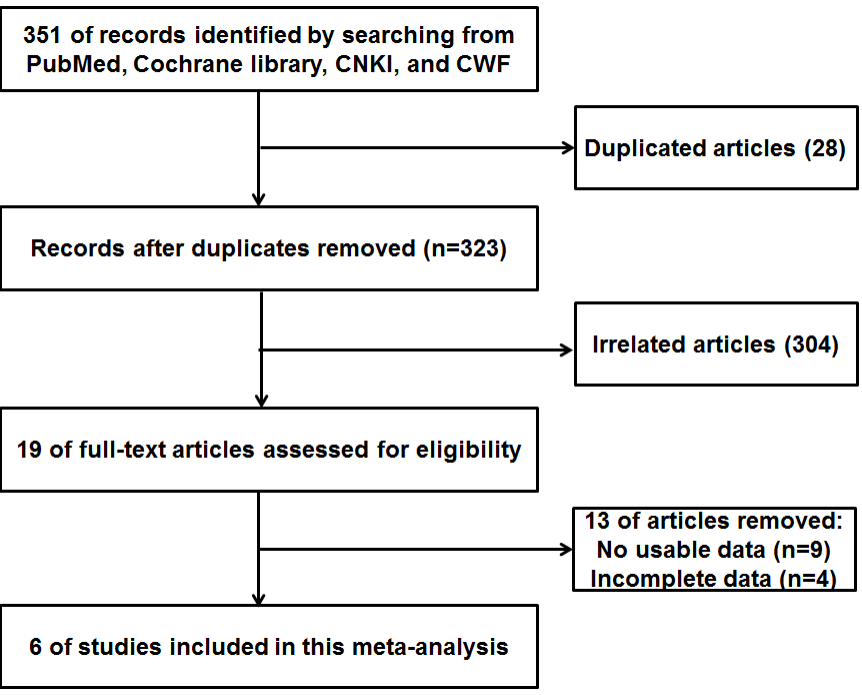
The flow diagram of this meta-analysis

As shown in the flow diagram (Figure 1), 351 potentially relevant articles were searched in the databases. After a detailed evaluation, 6 studies [18, 19, 21–24] were included in the current meta-analysis. The main characteristics of included studies were summarized in Table 1 ranging from 2003 to 2015. The total number of patients and controls were 578 and 562, respectively. Among the 6 studies, 3 studies were conducted in China [18, 23, 24], 1 in India [21], 1in Belgium [19], 1 in Egypt [22], which means three studies were conducted in Chinese populations, three studies were conducted in non-Chinese population. All the included studies used urine sediment as specimens and used reverse transcription polymerase chain reaction (RT-PCR) or quantitative RT-PCR method to determine the expression of UCA1 in urine sediment.

**Table 1:** Characteristics of the included studies

Study ID	Country	Ethnicity	Sample size		Cancer	Specimen	Method	Diagnostic power			
			Case	Control				TP	FP	FN	TN
Wang XS, 2006	China	Chinese	94	85	BC	urine	RT-PCR	76	7	18	78
Zhang Z, 2012	China	Chinese	180	144	BC	urine	RT-PCR	152	11	28	133
Li F, 2012	China	Chinese	24	50	BC	urine	qRT-PCR	21	20	3	30
Srivastava AK, 2014	India	non-Chinese	117	74	BC	urine	qRT-PCR	93	15	24	59
Milowich D, 2015	Belgium	non-Chinese	69	93	BC	urine	RT-PCR	48	27	21	66
Eissa S, 2015	Egypt	non-Chinese	94	116	BC	urine	qRT-PCR	86	4	8	112

BC: bladder carcinoma; TP: true positive; FP: false positive; TN: true negative; FN: false negative

### Quality assessment of studies

**Figure 2:**
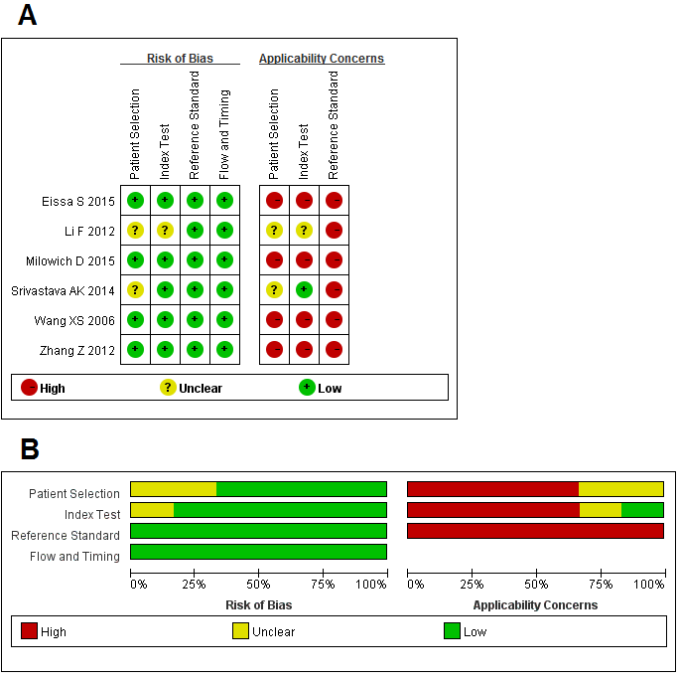
Quality assessments of included studies by using the QUADAS-2 tool **A**. risk of bias summary: review authors’ judgments about each risk of bias item for each included study; **B**. risk of bias graph: review authors’ judgments about each item presented as percentages across all included studies.

The results of the QUADAS-2 study quality assessment were shown in Figure 2. The majority of all included articles in current meta-analysis met most items in QUADAS-2, suggesting that the overall quality of included studies were of moderate-high.

### Diagnostic accuracy

**Figure 3:**
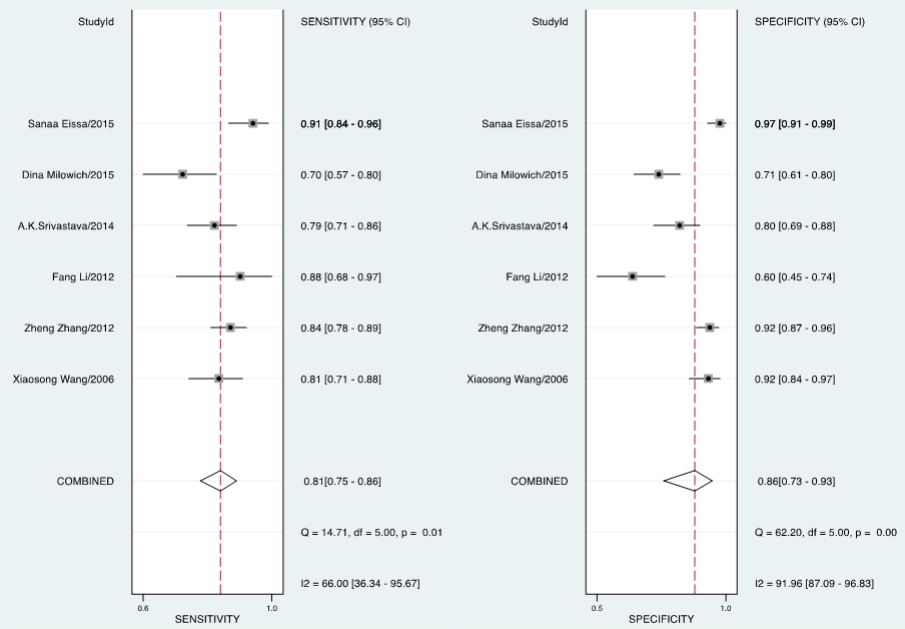
Forest plots of the sensitivity and specificity for UCA1 in the diagnosis of bladder cancer

**Figure 4:**
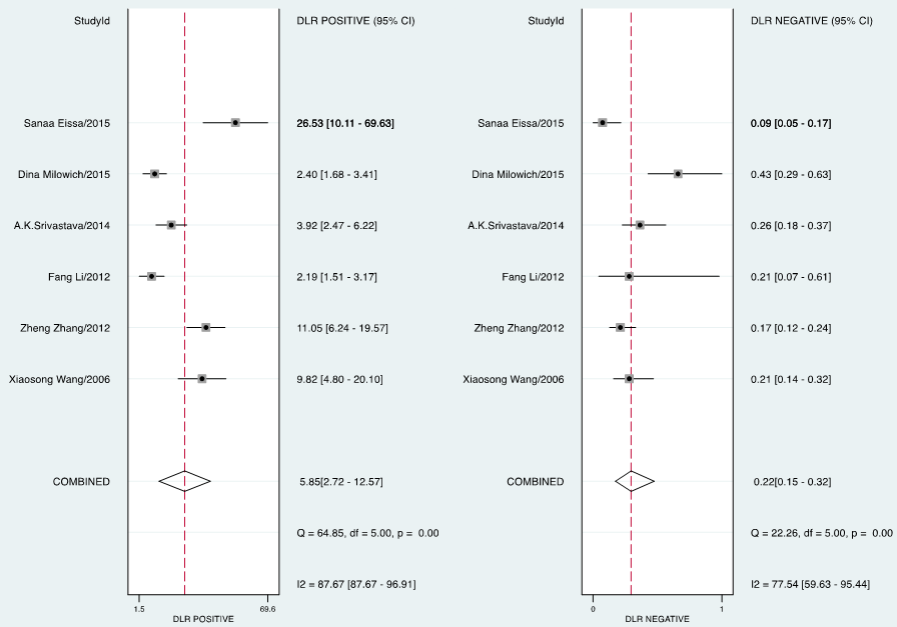
Forest plots of estimated positive likelihood ratio (PLR) and negative likelihood ratio (NLR) for urine UCA1 in the diagnosis of bladder cancer

**Figure 5:**
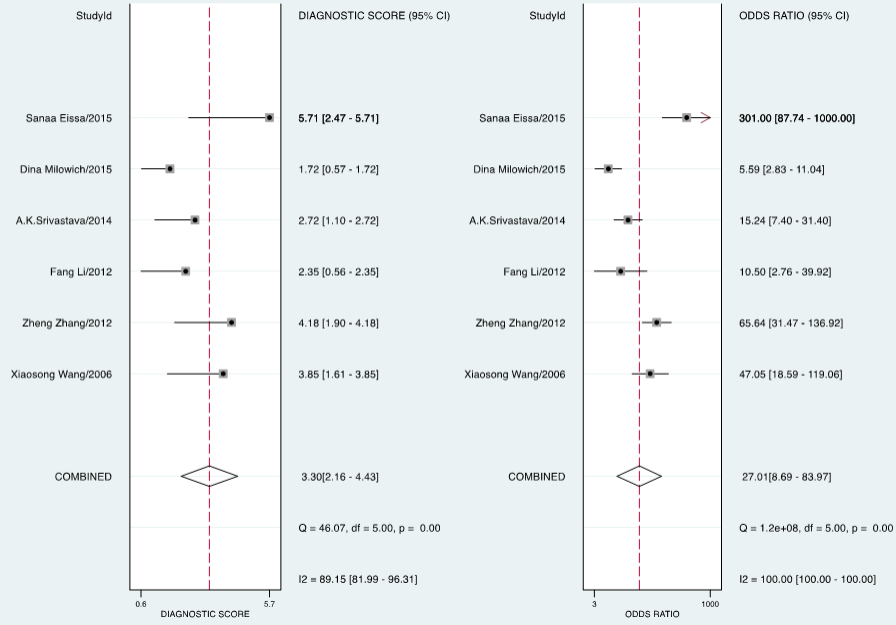
Forest plots of estimated pooled diagnostic odds ratio (DOR) for urine UCA1 in the diagnosis of bladder cancer

**Figure 6:**
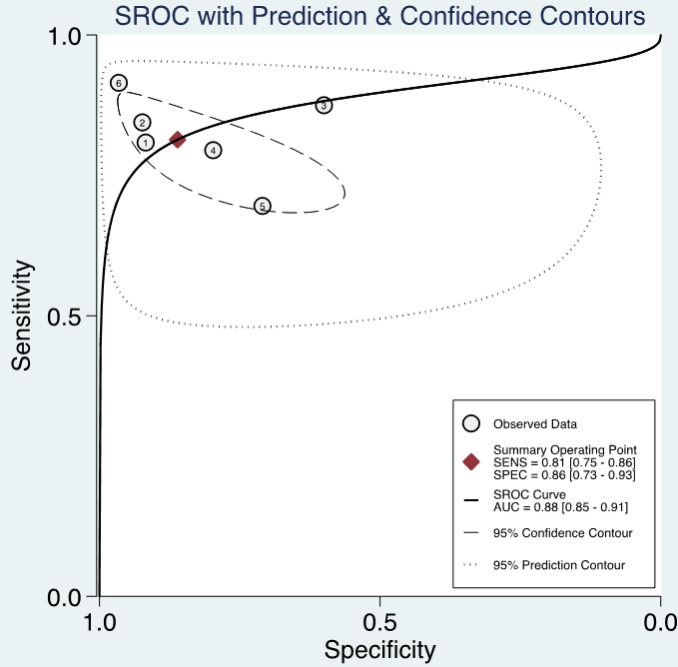
Summary receiver operating characteristic graph of included studies

**Figure 7:**
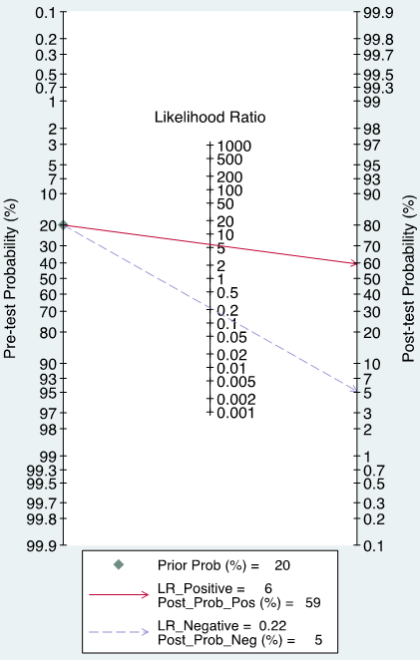
Fagan's nomogram for calculation of post-test probabilities

The forest plot of data from included articles on sensitivity and specificity for UCA1 assay in diagnosing bladder cancer is shown in Figure 3. Significant heterogeneity was found for both sensitivity (*I*^2^ = 66%, 95% CI = 36.34%-95.67%) and specificity (*I*^2^ = 91.96%, 95% CI = 87.09%-96.83%). Therefore, the random effects model was applied for the further analysis. Overall, the sensitivity and specificity for the pooled data were 0.81 (95% CI = 0.75-0.86) and 0.86 (95% CI = 0.73-0.93), respectively. In addition, the pooled PLR was 5.85 (95% CI = 2.72-12.57), the NLR was 0.22 (95% CI = 0.15-0.32), and the DOR was 27.01 (95% CI = 8.69-83.97) (Figure 4 and 5). The SROC curve for the 6 included studies in shown in Figure 6. The AUC of urine UCA1 tests was 0.88 (95% CI = 0.85-0.91), thereby implying a relatively high diagnostic value. To evaluate the clinical utility of the index test, a Fagan's Nomogram was performed to predict the increasing inerrability about a positive diagnosis by using the value of the test and it is used for estimating post-test probabilities (Figure 7).

### Meta-regression and subgroup analysis

**Figure 8:**
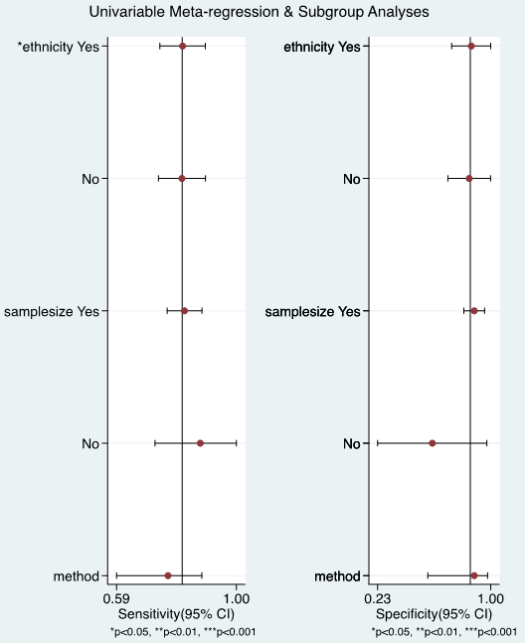
Univariable meta-regression and subgroup analysis

The sources of potential heterogeneity in sensitivity and specificity were explored by univariate meta-regression analysis and subgroup analysis. As shown in Figure 8, in the 3 Chinese studies, the pooled sensitivity and specificity was 0.81% (95% CI = 0.73-0.89) and 0.85% (95% CI = 0.71-1.00) respectively, and in 3 non-Chinese studies, the pooled sensitivity and specificity was 0.81 (95% CI = 0.74-0.89) and 0.87 (95% CI = 0.73-1.00) respectively. The ethnicity significantly accounted for the heterogeneity of sensitivity.

### Threshold effect

**Figure 9:**
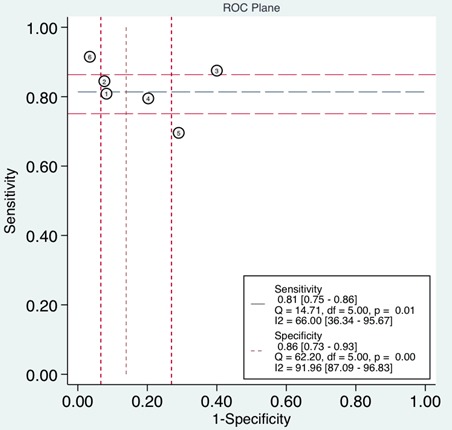
Receiver operating characteristics (ROC) space for the assessment of the threshold effect in UCA1 assays

Differences in cut-off values lead to the threshold effect. When there is a threshold effect, an inverse correlation is demonstrated among the sensitivity and specificity, leading to a typical ‘shoulder arm’ of the ROC plane distribution. Spearman correlation analysis is also suggests a strong positive correlation. In the current study, the representation of the sensitivity against the specificity of each study shown in an ROC plane (Figure 9), displayed a non-typical shoulder arm appearance, indicating the absence of the threshold effect. In addition, the calculated Spearman correlation coefficient value was -0.429 (*P* = 0.397), also suggesting that there was no threshold effect.

### Publication bias

**Figure 10:**
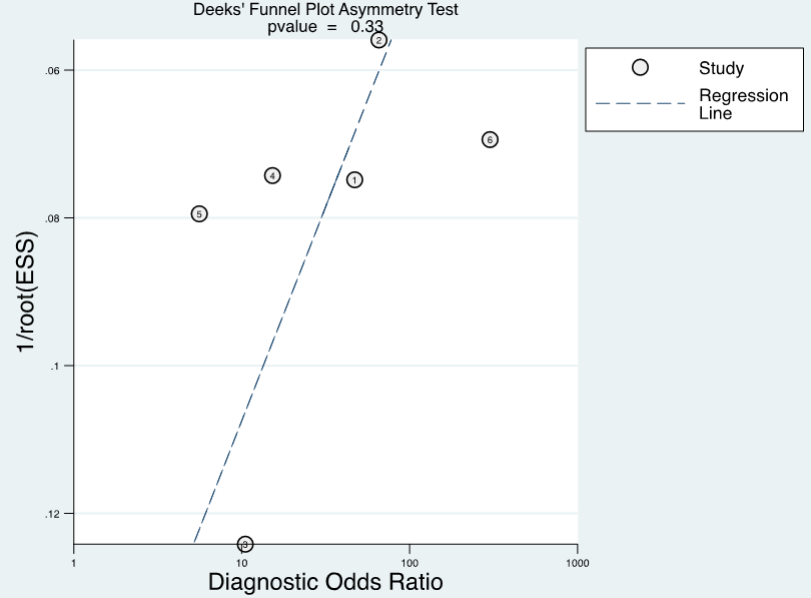
Deeks’ funnel plot asymmetry test for publication

In our meta-analysis, Deeks’ funnel plot asymmetry test was conducted to evaluate potential publication bias (Figure 10). No significant publication bias existed among the studies (*P* = 0.33).

## DISCUSSION

Bladder cancer is one of the most common male genitourinary tumors [25–27]. To date, although bladder cancer can be initial diagnosed by screening cystoscopy, random bladder biopsies, and voided urinary cytology, the first two methods are invasive and uncomfortable, and the low sensitivity and high variability of cytology creates a challenge that limits its application in the early diagnosis of bladder cancer due to inter-observer reproducibility [28–30]. Thus, searching a feasible, reliable, and minimally noninvasive method to detect new or recurrent bladder cancer is the need of the hour.

In current meta-analysis, we conducted the first diagnostic meta-analysis to assess the accuracy of urine UCA1 as a biomarker for bladder cancer. An AUC of 0.88 (95% CI, 0.85-0.91), with pooled SEN of 0.81 (95% CI, 0.75-0.86) and SPE of 0.86 (95% CI, 0.73-0.93) showed that UCA1 in voided urine sediment may be a promising biomarker to discriminate bladder cancer patients from normal bladder. As a prevalence-independent indicator, DOR value could indicate the degree of the association between diagnostic results and disease. The pooled DOR of 27.01 (95% CI, 8.69-83.97) suggested that the overall accuracy of UCA1 for the diagnosis of bladder cancer is credible.

The likelihood ratio (LR), including PLR and NLR, could also reflect the diagnostic accuracy. When positive likelihood ratio > 10 or negative likelihood ratio < 0.1, the likelihood of diagnosis or exclusion of a disease increased significantly. Nevertheless, in our meta-analysis, a pooled PLR of 5.85 (95% CI, 2.72-12.57) and NLR 0.22 (95% C, 0.15-0.32) indicated that patients with bladder cancer have an ~5.86-fold higher chance of testing positive using UCA1 compared with controls and 33% individuals with bladder cancer have an negative result.

LR and post-test probabilities are correlation with clinicians, due to they provide information about the likelihood of a patient with a positive or negative test actually exhibiting bladder cancer. From the Fagan's Nomogram, we found that when a pre-test probability of 20% was specified, the post-test probability positivity would raise to 59% with a positive likelihood ratio of 6, and the post-test probability negativity would decreased to 5% with a negative likelihood ratio was 0.22. These outcomes suggest a stable value for UCA1 in the diagnosis of bladder cancer.

Heterogeneity is a potential obstacle when interpreting the results for meta-analysis, which should be seriously considered [31]. Although rigorous approach has been adopted to retrieve documents, there was still potential heterogeneity in our current study. One source of heterogeneity is the threshold effect, which arises due to variable cut-off values adopted in different studies to determine whether the results were negative or positive. The ROC plane displayed a non-typical shoulder arm appearance, indicating the absence of the threshold effect. In addition, the calculated Spearman correlation coefficient value was -0.429 (*P* = 0.397), also suggesting that threshold effect is not the cause of heterogeneity. Then, we found that sample size and ethnicity may have partially led to such heterogeneity. Therefore, subgroup analyses were performed to evaluate the contribution of the factors above sources of potential heterogeneity in sensitivity and specificity was explored by univariate meta-regression analysis and subgroup analysis. Urine UCA1 has higher diagnostic accuracy for bladder cancer detection in non-Chinese persons than in Chinese persons, indicating that ethnicity may be one of the causes of heterogeneity. Due to there were insufficient eligible studies to fully elucidate the source of the heterogeneity, the possible reason for the heterogeneity need to be investigated in future studies.

The current study has several limitations. First, despite extensive literature search were performed, the number of included studies and sample sizes were small, which may restrict our ability to evaluate the accuracy of urine UCA1. Second, we could not determine the ideal cut-off value for urine UCA1 test, due to different cut-off values were adopted in each study. Third, this meta-analysis was a retrospective analysis, which may limit the conclusion due to selection bias. Fourth, only articles published in English or Chinese were enrolled in our meta-analysis, which may cause inevitable bias.

Despite these limitations, the present evidence suggests that urine UCA1 is potential to be a diagnosis biomarker for bladder cancer, due to this non-invasive method has good overall diagnostic performance. However, large-scale and comprehensive studies must be performed in the future to validate this finding.

## MATERIALS AND METHODS

### Search strategy

This meta-analysis as conducted under the diagnostic meta-analysis guidelines [32]. Studies regarding the diagnostic value of UCA1 in detecting bladder cancer were searched in PubMed, Cochrane library, Chinese Wan Fang and the China National Knowledge Infrastructure (CNKI) databases up to December 2016. Both MeSH terms and free-text words were used in the search strategy to increase sensitivity. The following search keywords were used in combination: “UCA1” or “urothelial carcinoma-associated 1”, “bladder cancer” or “bladder carcinoma” or “carcinoma of urinary bladder”. In addition, references of all articles in these eligible studies were also read to identify additional relevant literature.

### Inclusion and exclusion criteria

The inclusion criteria were listed as follows: (1) articles were association between UCA1 and bladder cancer; (2) a diagnostic standard of bladder cancer was included; (3) sufficient data (true positive, false positive, false negative and true negative) for calculating sensitivity and specificity; (4) studies should base on humans; (5) studies were published in English or Chinese. Exclusion criteria were as follows: (1) studies without usable or overlapping data; (2) reviews, letters, case report and conference abstracts; (3) insufficient data for calculating sensitivity and specificity.

### Data extraction

Two investigators (Xiangrong Cui and Xuan Jing) retrieved the eligible publications independently. Any disagreement between the two investigators was determined through a discussion with a third investigator. The following data were extracted: first author, year of publication, country, ethnicity of patients, sample size, type of specimen, true and false positive and negative.

### Statistical analysis

For the diagnostic meta-analysis, the accuracy indicator include the pooled sensitivity (SEN), pooled specificity (SPE), positive likelihood ratio (PLR), negative likelihood ration (NLR), diagnostic odds ratio (DOR), and their 95% confidence interval (CI) were calculated using the random-effect model. PLR was on behalf of the odds of positive test results of bladder cancer patients, while NLR reflected the odds of positive results in those without bladder cancer. DOR was the outcome of the combination of PLR and NLR (DOR = PLR/NLR). Simultaneously, the summary receiver operator characteristic (SROC) curve was created and the under the SROC curve (AUC) was calculated [33]. The analysis of diagnostic accuracy was pursuant to a SROC curve and the AUC of the SROC. In addition, the *I*^2^ and *Q* test were performed to evaluate heterogeneity in SEN and SPE among included studies. If the tests show a *P* < 0.1 or *I*^2^ > 50%, the existence of significant heterogeneity would be verified, and then the random-effect model was employed. If not, the fixed-effect model was more appropriate [34, 35]. Subsequently, meta-regression and subgroup analyses were conducted to explore potential sources of between-study heterogeneity. Furthermore, Deeks’ funnel plots were adopted to test the publication bias [36]. All statistical analyses were performed using RevMan 5.3 (Revman, the Cochrane Collaboration) and Stata 12.0 (Stata, College Station).
